# Molecular Chaperones in Osteosarcoma: Diagnosis and Therapeutic Issues

**DOI:** 10.3390/cells10040754

**Published:** 2021-03-30

**Authors:** Morgane Lallier, Louise Marchandet, Brice Moukengue, Celine Charrier, Marc Baud’huin, Franck Verrecchia, Benjamin Ory, François Lamoureux

**Affiliations:** 1 UMR1238, Phy-OS, Sarcomes Osseux et Remodelage des Tissus Calcifiés, INSERM, Université de Nantes, 44035 Nantes, France; morgane.lallier@univ-nantes.fr (M.L.); louise.marchandet@univ-nantes.fr (L.M.); bricemoukengue@yahoo.fr (B.M.); celine.charrier@univ-nantes.fr (C.C.); marc.baudhuin@univ-nantes.fr (M.B.); franck.verrecchia@univ-nantes.fr (F.V.); Benjamin.Ory@univ-nantes.fr (B.O.); 2 CHU Nantes, 44035 Nantes, France

**Keywords:** osteosarcoma, bone tumor, HSPs, HSF1

## Abstract

Osteosarcoma (OS) is the most common form of primary bone tumor affecting mainly children and young adults. Despite therapeutic progress, the 5-year survival rate is 70%, but it drops drastically to 30% for poor responders to therapies or for patients with metastases. Identifying new therapeutic targets is thus essential. Heat Shock Proteins (HSPs) are the main effectors of Heat Shock Response (HSR), the expression of which is induced by stressors. HSPs are a large family of proteins involved in the folding and maturation of other proteins in order to maintain proteostasis. HSP overexpression is observed in many cancers, including breast, prostate, colorectal, lung, and ovarian, as well as OS. In this article we reviewed the significant role played by HSPs in molecular mechanisms leading to OS development and progression. HSPs are directly involved in OS cell proliferation, apoptosis inhibition, migration, and drug resistance. We focused on HSP27, HSP60, HSP70 and HSP90 and summarized their potential clinical uses in OS as either biomarkers for diagnosis or therapeutic targets. Finally, based on different types of cancer, we consider the advantage of targeting heat shock factor 1 (HSF1), the major transcriptional regulator of HSPs in OS.

## 1. Introduction

### 1.1. General Description

Osteosarcoma (OS) is the most common form of primary bone tumor affecting mainly children and young adults. The main peak in incidence of this disease is 18 years. Worldwide, the incidence of OS is 1 to 3 cases annually per million people [1,2]. OS is characterized by an anarchic degradation or formation of bone tissue causing acute locoregional pain.

The causes of the disease are poorly understood; however, tumor location—mainly in long bone metaphyses near the growth plate—suggests that rapid growth in the subject could be a risk factor [3]. A few hereditary genetic predispositions also seem to play a role in OS development: 12% of patients with Li-Fraumeni syndrome, the result of a mutation in the *TP53* gene, have been shown to develop OS. Likewise, mutations in the tumor suppressor gene *RB1*, a key regulator of cell cycle progression, increases incidence of the disease in subjects by a hundred [4].

### 1.2. Current Support and Limitations

Clinical care has undergone a few changes in the past 40 years. It is currently based on neoadjuvant polychemotherapy (high-dose methotrexate (MTX), doxorubicin (DOX), and cisplatin (CIS)) followed by surgery for *en bloc* resection of the tumor. After this, adjuvant polychemotherapy, adapted according to response to the first chemotherapy, can be administered [5]. The boom in chemotherapy has increased the 5-year survival rate to 70% for good responders to treatment. Increasing doses of adjuvant chemotherapy and administration of new combinations of drugs, in poor responders, do not show any increase in their overall survival but considerably increase the side effects, as well as the risk of relapses [6]. Thus, the 5-year survival rate is only 30% for poor responders to chemotherapy and for those with pulmonary metastases at time of diagnosis [7]. The sum of the observed side effects, added to numerous resistance phenomena, shows the limitations of this therapeutic strategy and the need to develop new targeted approaches.

### 1.3. Heat Shock Proteins as Potential New Therapeutic Targets in OS

One of characteristics that differentiate cancer cells from healthy cells is their permanent exposure to high levels of stress. These stresses are induced by phenomena such as nutrient or oxygen deprivation, genetic mutations or even exposure to therapeutic agents, leading to degradation of macromolecules essential to the life of the cells, such as DNA, RNA, or proteins. To protect themselves against protein degradation, cancer cells take advantage of a known physiological mechanism used in healthy cells to fight against various environmental attacks: the Heat Shock Response (HSR). Heat Shock Proteins (HSPs) are the main actor in the HSR. Cancer cells have a higher need for HSPs than normal cells to maintain their survival. This hijacking of a physiological phenomenon has been described as cancer cells’ “non-oncogene addiction” [8]. It has been demonstrated that HSP inhibition induces cancer cell death [9,10].

HSPs are a large family of molecular chaperones classified according to their molecular weights. The 6 major HSP groups are small HSPs including HSP27 (HSPB1), HSP40 (DNAJ), HSP60 (HSPD1), HSP70 (HSPA), HSP90 (HSPC), and large HSPs [10,11,12]. These proteins are encoded by different genes and, except in the case of small HSPs, are ATP-dependent proteins with ATPase activity [13]. Their role is to interact with immature or abnormal proteins, inhibiting their degradation or abnormal aggregation, making genuine protein homeostasis possible. HSPs are proteins that are highly regulated to respond rapidly to protein damage induced by different stresses.

HSPs are overexpressed in a wide range of tumor types including breast, prostate, colorectal, lung cancers and OS [14,15], and their elevated expression levels are associated with both poor prognoses for patients and increased resistance to therapies [12,16,17]. HSPs are studied for their potential use as prognostic markers in OS and as therapeutic targets that could be combined with conventional therapies for poor responders to treatment. In this review manuscript, we focused on the relationship between OS and the four best-studied HSP members to date: HSP27, HSP60, HSP70 and HSP90, and their major transcriptional regulator HSF1.

## 2. The Biology of HSPs

### 2.1. HSPs in the HSR

Physiologically, the HSR acts as a response to several types of stress, whether thermal (temperature rise above 37 °C in humans), oxidative (increased rate of reactive oxygen species (ROS) in the cell), or caused by heavy metals, toxins or an infectious phenomenon [18]. The importance of this mechanism is demonstrated by its high conservation, observed from yeast to humans [19,20,21]. The HSR works to maintain proteostasis (protein homeostasis) by allowing proteins to maintain proper folding. A sustained increase in temperature effectively alters the three-dimensional conformation of proteins, thus affecting their function. A secondary effect of denaturing proteins is that it exposes hydrophobic groups of peptidic chains, leading to the formation of aggregates that are harmful to cell homeostasis [22]. Heat stress can also lead to the destruction of microtubules, causing a disruption in intracellular signalization, which relies on these cytoskeleton proteins [23,24]. An HSR’s action is mediated by chaperone proteins (Heat Shock Proteins, HSPs). Thanks to HSPs, poorly conformed proteins are prevented from being degraded by the proteasome or the formation of amorphous aggregates that can induce cell death [25]. Proteins, as the main cellular effectors, thus play the role of guardian, and this indirectly involves HSPs in many key biological processes for the cell.

### 2.2. General HSP Regulation

**Figure 1:**
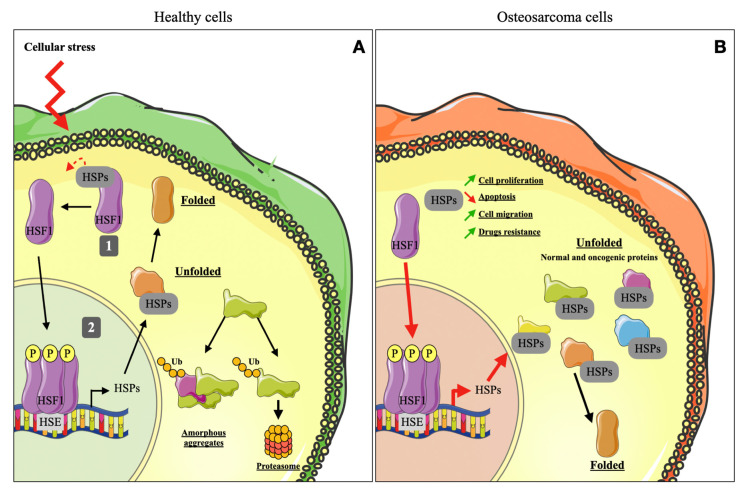
HSR and HSP functions in a healthy cell and OS cell. (**A**) Under physiological conditions, HSF1 is complexed with HSPs and is located in the cell cytoplasm (1). During cellular stress, HSF1 is phosphorylated, trimerized and translocated into the nucleus where it binds to the HSE motif and initiates transcription of HSPs (2). Some unfolded proteins are taken over by HSPs to be refolded. Others are ubiquitinylated and degraded by the proteasome. Accumulation of unfolded proteins leads to amorphous aggregations, compromising the cell’s survival. (**B**) In a cancer cell, HSF1 is over-activated and initiates the transcription of numerous HSPs, which take over normal and oncogenic unfolded proteins and refold them, thereby averting proteomic instability. Furthermore, HSPs are directly involved in different mechanisms that promote cell survival, such as cell proliferation and migration, apoptosis and drug resistance. HSE: Heat Shock Element; HSF1: Heat Shock Factor 1; HSPs: Heat Shock Proteins; Ub: Ubiquitin; P: Phosphorylation.

HSF1 (Heat Shock Factor 1) is the main transcription factor in the response to stress. Inhibiting HSF1 in mice prevents HSP induction and makes cells vulnerable to proteotoxic stress [26]. HSP expression is thus highly dependent on HSF1 regulation (Figure 1). This regulation is affected by both protein interactions and post-translational modifications. The first level of regulation of HSF1 is in a feedback loop carried out by HSPs. HSF1 spontaneously interacts with a complex composed of chaperone proteins HSP90, HSP70 and HSP40, preventing it from binding to DNA. During an episode of stress, the cell is no longer able to ensure protein homeostasis with the pool of chaperones it has. Badly conforming proteins accumulate in the cytoplasm of cells, subsequently inducing competition between the malformed proteins and HSF1 for HSPs. HSF1′s binding to HSP breaks, allowing free HSF1 to homotrimerize and bind DNA into specific sequences called heat shock elements (HSEs), cis-acting sequences that are located upstream of HSP genes. HSF1 binding to HSE induces HSP gene expression. When cellular stress ceases, the number of proteins labelled “client” in the HSPs decreases. The HSP–HSF1 complex is reformed, preventing its later activation [27,28].

In addition, hyperphosphorylation of HSF1 has been shown to be essential for its activation. HSF1 undergoes numerous activating, as well as inhibiting, phosphorylation phases. However, serine 326 phosphorylation is the essential modification for HSF1 activation in a stress response. This modification is carried out in particular by the RAS/MAPK signalization pathway. This pathway is mutated in 30% of human cancers and governs a large number of cellular processes, such as proliferation, differentiation, transcription and translation, as well as cell survival [29]. Thus, cancerous cell mutations and mechanisms also contribute to HSP over-expression.

### 2.3. HSPs Promote Carcinogenesis in Cancer Cells

HSPs indirectly promote carcinogenesis in different ways. First, they guard the cancer proteome by stabilizing oncoproteins. As chaperone proteins, HSPs maintain the three-dimensional conformation and stability of many cellular proteins, including oncoproteins. For example, oncogenes implicated in anarchic cellular proliferation such as epidermal growth factor (EGFR), platelet derived growth factor (PDGF), fibroblast growth factor (FGF), human epidermal growth factor receptor-2 (HER2) and HER3, and other proteins such as RAS, PI-3 Kinase or PTEN, have relatively unstable conformations and are, incidentally, highly dependent on HSP90 chaperoning [30,31,32].

Many oncoproteins mutated de novo in cancer cells also tend to be stabilized by HSPs such as BCR–ABL or mutated TP53. In addition, HSPs work by suppressing protein aggregates and amyloidogenesis (protein aggregates enriched in beta sheets often associated with the development of neurodegenerative diseases), deposits that physiologically used to lead to cell death [29].

HSPs also promote carcinogenesis directly by regulating cell proliferation and inhibiting apoptosis, resulting in the promotion of resistance mechanisms. As a first example, HSP27 is involved in cell cycle progression, repressing cell cycle inhibitory proteins E2F-4 and p130 [33]. HSPs also help cancer cells escape apoptosis by blocking protein intermediates in the signaling pathways governing programmed cell death [34,35,36]. HSP90 is also known to stabilize telomerase in certain forms of cancer [37]. Furthermore, HSPs are described as promoting invasion and metastasis. HSP27 is effectively necessary for invasion and metastasis triggered by hepatocyte growth factor (HGF) [38] and is involved in the regulation of matrix metalloproteinase 9 (MMP9), a major invasion mediator [39]. HSP90 or HSP70 imbalance has also been shown to disturb key proteins involved in migration and invasion, such as focal adhesion kinase (FAK) and the Wiskott–Aldrich syndrome protein family member 3 (WASF3) [40,41].

## 3. The Different Roles Played by HSPs in OS and Their Therapeutic Benefits

### 3.1. HSP27

#### 3.1.1. Structure

**Figure 2:**
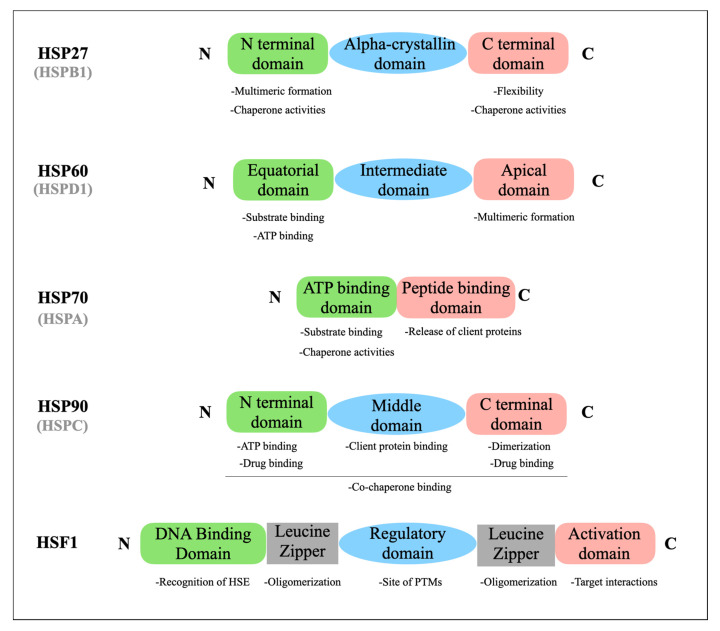
Schematic structure of HSP27 (HSPB1), HSP60 (HSPD1), HSP70 (HSPA), HSP90 (HSPC) and HSF1. The main functions of each domain are specified. HSP: Heat Shock Protein; HSF1: Heat Shock Factor 1; HSE: Heat Shock Element; PTMs: Post Translational Modifications.

HSP27 (HSPB1) belongs to the family of small HSPs, which includes proteins from 15 to 30 kDa. HSP27 is composed of 205 amino acids and encoded by the three exons of the human *HSPB1* gene, located on chromosome 7q11.23 [11,42]. The structure of HSP27 is composed of 3 parts: an N-terminal domain (essential for HSP27 multimeric formation and chaperone activity), an alpha-crystallin domain and a C-terminal domain (involved in the formation of a flexible structure that is important for chaperone functions) (Figure 2). HSP27 is present in both cytoplasm and the nucleus [43].

#### 3.1.2. Regulation

The *HSPB1* gene contains two functional HSE binding sites, the first located −200 bp upstream of exon 1, and the second within the first intron. These sites bind HSF1 and HSF2 respectively [42,44,45]. HSP27 expression is induced by the binding of HSF1 to HSE after heat shock [45,46]. HSP27 is also exposed to different post translational modifications affecting its functions. HSP27 is particularly phosphorylated at serine 15, serine 78 and serine 82 in response to stress [47]. Its phosphorylation is catalyzed by mitogen-activated protein kinase-activated protein kinases (MAPKAPK) 2 and 3, which are activated through upstream phosphorylation of p38 mitogen-activated protein kinase (MAPK). The unphosphorylated form of HSP27 can form multimers reaching up to the 800 kDa threshold required to activate its chaperone function [48]. This chaperone activity is regulated by serine 15, serine 78 and serine 82 phosphorylation that induces conformational changes in the protein, making its oligomeric size smaller. It has been demonstrated with insulin and α-lactalbumin that the most active chaperone is the dimeric form of HSP27 [49]. Through its smaller size, HSP27 can also interact with protein partners including β-catenin, histone deacetylase 6 (HDAC6), procaspase-3, and the signal transducer and activator of transcription 2 (STAT2) [50].

#### 3.1.3. The Role of HSP27 in Cancer

Like all other HSPs, HSP27′s primary role is to maintain cellular homeostasis, promoting cell survival under lethal conditions. However, the initial function of HSPs is corrupted in tumor cells, where HSP27 is involved in tumor development by promoting cell survival, motility, and drug resistance mechanisms. Over-expression of HSP27 is closely related to tumorigenesis, metastasis and invasiveness in breast, prostate and ovarian cancers [38,51,52]. HSP27 expression is also correlated with poor prognosis in prostate cancer, intrahepatic cholangiocarcinoma and rectal cancer [52,53]. HSP27 over-expression is moreover associated with chemotherapy drug resistance in tumor cells. In fact, in breast cancer, HSP27 inhibits doxorubicin-induced apoptosis by regulating topoisomerase II expression. [54]. This HSP27 involvement in tumorigenesis is related to its chaperone functions but also to its direct involvement in the apoptosis pathway [55]. HSP27 is described as inhibiting both intrinsic and extrinsic apoptosis pathways through binding to cytochrome C or death domain associated protein (DAXX) [56,57]. HSP27 is involved in the promotion of cancer drug resistance by its interaction with protein kinase C delta type (PKC δ) [58]. HSP27 might also protect cancer cells from etoposide or TNF-alpha-induced apoptosis by increasing the activity of nuclear factor-kappa B (NF–kB) [55,59]. HSP27 is also involved in the regulation of cytoskeleton dynamics thanks to its ability to interact with the microtubule actin protein. This can help to promote cell invasion and survival [60]. Finally, HSP27 overexpression is observed in many cancers [61,62,63] and is associated with tumor metastasis and a poor prognosis for patients [12].

#### 3.1.4. HSP27 in OS

**Figure 3:**
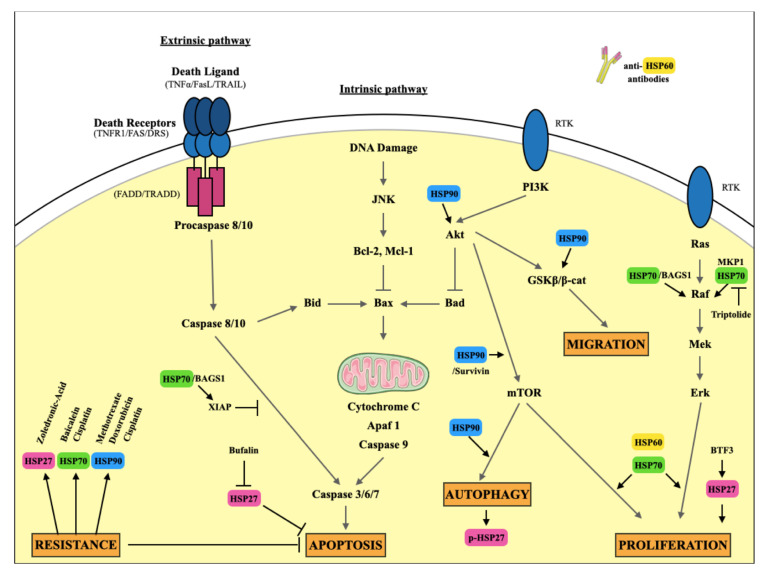
Overview of the major signaling pathways involving HSPs in cancer promotion in OS. HSP27, HSP60, HSP70 and HSP90 are involved in different ways in OS cell proliferation, apoptosis, migration, autophagy and resistance. BAG: Bcl-2 associated athanogene; XIAP: X-linked inhibitor of apoptosis protein; Apaf: Apoptotic peptidase factor; Bid: BH3 interacting-domain death agonist; Bax: Bcl-2 associated X protein; Bad: Bcl-2 associated death promoter; JNK: c-Jun N-terminal kinases; Bcl: B-cell lymphoma; Mcl: Induced myeloid leukemia cell differentiation protein; Akt: Protein kinase B; PI3k: Phosphoinositide 3-kinase; RTK: Receptor tyrosine kinase; GSK: Glycogen synthase kinase; MKP: MAPK phosphatases; Raf: Rapid accelerated fibrosarcoma; Mek: Mitogen-activated protein kinase kinase; Erk: Extracellular signal regulated kinases; TNF: Tumor necrosis factor; TRAIL: Tumor necrosis factor related apoptosis inducing ligand; FADD: Fas associated protein with death domain; TRADD: Tumor necrosis factor receptor type 1-associated death domain protein.

HSP27 is overexpressed in 22% of OS biopsies [64]. It promotes OS development and progression by playing a role in OS proliferation, apoptosis, migration, and resistance mechanisms against conventional therapies (Figure 3). Liu et al., demonstrate that HSP27 seems to be involved in OS proliferation through basic transcription factor 3 (BTF3) regulation. BTF3 knockdown down-regulates HSP27 as well as STAT3, S6 ribosomal protein and SAPK/JNK2. BTF3 is a transcription factor that can form a stable complex with RNA polymerase II and is required for initiation of transcription. BTF3 initiates development of various cancer types including gastric and prostate cancer [65]. Inhibition of BTF3 significantly inhibited cell proliferation and enhanced apoptosis. This suggests that HSP27 is activated by BTF3 and involved in some of these functions [66].

HSP27 is also implicated in the motility of OS cells by reducing autocrine motility factor (AMF) expression under hyperthermia [67]. AMF, by regulating tumor cell motility, plays an important role in the development of metastasis. Indeed, AMF inhibition induces mesenchymal to epithelial transition (MET) and suppresses tumor growth and pulmonary metastasis in OS mice [68].

HSP27 is described as playing an anti-apoptotic role in OS. Bufalin (Bufadienolide, demonstrated anti-tumor activity in various cancers) induces apoptosis in a dose-dependent manner by down-regulating HSP27 expression in MTX sensitive and resistant OS cells [69]. HSP27 downregulation induced by Bufalin decreases p-Akt and nuclear NF–kB p65, reduces HSP27 interaction with cytochrome C and induces PARP cleavage. Moreover, HSP27 over-expression reverses Bufalin effects in OS cells suggesting that that expression level of HSP27 could predict OS cell response to Bufalin [69]. HSP27 is also involved in OS cells resistance to Zoledronic acid (ZOL). Different studies demonstrates that ZOL directly affects OS cell proliferation and survival [70,71]. While HSP27 is upregulated in ZOL-resistant cell lines, HSP27 silencing increased sensitivity to ZOL in resistant cells. This resistance mechanism seems to be directly related to the anti-apoptosis role of HSP27 in OS cells [72].

Finally, HSP27 is described as the strongest biomarker in OS. Its overexpression in tumor is associated with poor prognosis for patients, distant metastases, and high risk of relapse and death [64,73,74,75], and its absence seems to be associated with a favorable prognosis in canine OS [76]. Phosphorylated HSP27 could be used as a predictive biomarker in anticancer drug treatment, including autophagy. Expression of p-HSP27 correlates with the response to drug-induced autophagy (gemcitabine and 9-NC), so that p-HSP27 expression could have an impact on the effect of autophagy on cell viability after treatment with anticancer drugs known to induce autophagy [77].

Thus, due to its anti-apoptotic and tumorigenic properties, HSP27 could be a potential therapeutic target for OS.

#### 3.1.5. Targeting HSP27 in OS

A few molecules tested in vitro target HSP27 in OS, for example: KRIBB-3, an inhibitor of PKC-dependent phosphorylation of HSP27 by direct binding to HSP27. KRIBB-3 also inhibits microtubule polymerization [78]. Several in vitro and in vivo studies have shown that KRIBB-3 enhances the efficiency of other anticancer agents and activates apoptosis in cancer cells [78,79]. In OS cells, it has been used under hyperthermia conditions and induces AMF expression, which can promote the migration of OS cells [67]. Bufalin induced apoptosis in OS cells by significantly reducing endogenous levels of HSP27 in a dose-dependent manner, while its level of mRNA remained remarkably unchanged. Treatment of MG132, a proteasome inhibitor, restored HSP27 levels, suggesting that Bufalin might be able to induce the degradation of HSP27 in OS cells [69].

### 3.2. HSP60

#### 3.2.1. Structure

HSP60 (HSPD1) is mainly located in the mitochondria of eucaryote cells, but also occurs in cytosol, on the cell surface, in the extracellular space, and in peripheral blood [11,80]. HSP60 is composed of 573 amino acids and contains three main domains: the apical, intermediate, and equatorial domains (Figure 2). The apical domain binds the substrates and is implicated in ATP turnover. The intermediate domain connects the apical with the equatorial domain. Finally, the equatorial domain is involved in the different conformations of HSP60 [81].

#### 3.2.2. Regulation

HSP60 interacts with its co-chaperone HSP10 in mitochondria to maintain homeostasis. The genes that encode HSP60 and HSP10 are juxtaposed on chromosome 2 and share a common bidirectional promoter [82]. HSP60 is subject to different kinds of PTM, such as phosphorylation, O-GlcNAcylation, N-glycosylation, nitration, C-nitrosylation, methylation, oxidation, or ubiquitination. These PTMs can affect HSP60′s biological function. For example, nitration on tyrosine 222 and 226 inhibits its folding activity [83].

#### 3.2.3. The Role of HSP60 in Cancer

HSP60 expression is increased in various cancers, including colorectal, gastric, pancreatic, prostate or breast [10]. It has been shown to promote the transformation, angiogenesis and metastasis of cancer cells [84]. HSP60 is involved in cell survival by regulating different proteins such as IkB and clusterin (CLU). In fact, it is able to interact and regulate the activity of IkB kinase, which is involved in NF–kB-dependent cell survival in HeLa cells [85]. In neuroblastoma cells, HSP60 interacts and inhibits intracellular CLU, thus promoting cell survival. CLU exerts tumor suppressive functions by inhibiting the activity of NF–kB [86]. HSP60 is also involved in regulating apoptosis and is associated with a component of the mitochondrial permeability transition pore: cyclophilin D (CypD). This interaction only occurs in the context of tumor cells and it has been observed that HSP60 inhibition affects CypD-dependent mitochondrial permeability transition and caspase-dependent apoptosis, inducing the suppression of glioblastoma intracranial cell growth in vivo [87]. HSP60 overexpression also induces metastatic phenotypes in different cancers. Part of its pro-metastatic role could be explained by its interaction with ß-catenin, observed in cancer cells [88]. It has also been observed that HSP60 seems to be involved in drug resistance mechanisms as it is overexpressed in CIS-resistant ovarian and bladder cancer cells compared to sensitive cells [89,90].

#### 3.2.4. HSP60 in OS

HSP60 is highly expressed in the tumors of OS patients [64,74], but to date no correlation has been established with clinicopathological parameters. However, Trieb et al. observed that 43% of OS patients present high levels of anti-HSP60 antibodies while only 6% of healthy controls have such high levels [91]. This data suggests the potential diagnostic role of HSP60 in OS. It has thus been demonstrated, using RNA interference, that HSP60 inhibition stopped U2OS cell proliferation [92]. HSP60 downregulation also reduces cells proliferation and induces apoptosis in canine OS cell lines [93], suggesting the potential therapeutic interest to target HSP60 in cancer including OS [84].

#### 3.2.5. Targeting HSP60 in OS

Only RNA interference has been used in OS to downregulate HSP60 as it inhibits cell proliferation (Figure 3) [92]. However, geldanamycin, an HSP90 inhibitor, induces a subsequent upregulation of HSP70 and HSP90, as well as an increase in HSP60 expression that could lead to drug resistance in OS cells. HSP60 upregulation is accompanied by a simultaneous loss of the hyperacetylated HSP60 mitochondrial protein pool, resulting in decreased viability and increased cancer cell death [94]. These observations underline the importance of considering HSP60 as an interesting new therapeutic target in OS.

### 3.3. HSP70

#### 3.3.1. Structure

HSP70 (HSPA) contains two major domains, each with a different function [11]. The first contains a peptide binding domain (PBD), responsible for substrate binding and refolding. The second contains an amino-terminal ATPase domain (ABD) that facilitates release of client proteins after ATP hydrolysis (Figure 2). HSP70 binds directly to exposed hydrophobic residues on unfolded proteins [95]. There are 13 HSP70 isoforms present in different cell compartments, such as cytosol, the nucleus, lysosomes, endoplasmic reticulum and mitochondria [96]. HSP70 family members are highly homologous, but only a few isoforms (HspA1A, HspA1B, HspA6, HspA7, HspA14) show stress-inducible expression and are regulated by HSF1 transcription factor [97,98].

#### 3.3.2. Regulation

HSP70 is regulated by different co-chaperone proteins that modulate its chaperone function. These co-chaperones can be classified in different categories. The J-domain co-chaperone group is represented by HSP40 which binds to HSP70 ABD and stimulates the ATPase activity of HSP70, triggering transition to ADP-bound states [99].

In contrast to HSP40, the co-chaperones of the nucleotide exchange factor (NEF) group catalyze the release of ADP, resulting in ATP and substrate release. This group includes Bcl2-associated anthanogen (BAG), HSP110 and HSPBP1 co-chaperones [100].

Finally, the tetratrico-peptide repeat (TRP) domain co-chaperone group consists of two proteins, HOP and CHIP, acting in a complementary manner. These proteins bind to the C-terminal EEVD motif, present in both HSP70 and HSP90. HOP mediates the interaction between HSP70 and HSP90, allowing the passage of client proteins. Substrates that spend too much time bound to HSP70, thus remaining folded by HSP90, are targeted to be degraded by the ubiquitin-proteasome system by the protein CHIP [101,102].

#### 3.3.3. The Role of HSP70 in Cancer

Like other HSPs, HSP70 is overexpressed in many cancers, allowing the survival of most cancer cells [103]. HSP70 downregulation strongly decreases tumorigenicity in experimental models [104]. Beyond protecting cells from stress thanks to its chaperone activity, HSP70 is involved in many biological processes that directly promote tumorigenicity such as cell proliferation, apoptosis, angiogenesis, cell migration, and drug resistance. HSP70 interacts with actors from different signaling pathways implicated in cell proliferation, such as Akt/PKB. HSP70 binds and stabilizes Akt/PKB, a serine/threonine kinase that generates a survival signal in response to growth factor stimulation [105]. Moreover, HSP70 is shown to suppress apoptosis [106]. HSP70 is effectively involved in the extrinsic pathway of apoptosis, interacting with TRAIL-R1 and TRAIL-R2 and preventing DISC complex formation. HSP70 is also involved in the intrinsic pathway, inhibiting JNK and p38, thus preventing the translocation of Bax and Bid to the mitochondria [107,108]. In both ways, HSP70 blocks cytochrome c within the cytosol, and the formation of the apoptosome complex with Apaf-1 and caspase-9. HSP70 may also directly avoid the formation of the apoptosome by binding to Apaf-1 and preventing its association with procaspase-9 [34]. Probably because of its ability to inhibit apoptosis, HSP70 downregulation can reverse cancer cell drug resistance. It has been demonstrated that the BCR–ABL oncoprotein up-regulates HSP70 by binding the GATA–RE element to the HSP70 promoter region in chronic myelogenous leukemia (CML). HSP70 downregulation increases cell sensitivity to paclitaxel-induced apoptosis [109]. HSP70 is also involved in angiogenesis by binding and stabilizing hypoxia inducible factor-1 alpha (HIF-1α) in renal cell carcinoma. HIF-1α is a transcription factor that senses low oxygen availability and thus regulates expression of genes that play a role in angiogenesis [110]. HSP70 extracellular peptide complexes also seem to induce epithelial-mesenchymal transition (EMT), promoting migration in hepatocarcinoma cell lines through activation of the p38/MAPK signaling pathway. While extracellular HSP70 peptide complex activation reduces E-cadherin expression and induces alpha-smooth muscle actin (α-SMA) expression, inhibition of the p38/MAPK signaling pathway using SB-203580 inhibitor increases E-cadherin expression and decreases α-SMA expression [111,112]. In addition, HSP70 has been shown to serve as an effective vaccine, producing an anti-tumor immune response in humans. The vaccine, consisting of hydroxyapatite particles and autologous tumor cell membrane including HSP70 and HSP27, had a positive response in a few patients. Out the 20 patients injected, 25% exhibited stabilization in the progression of their cancer, 15% of patients presented a partial response, and 4 patients with recurrent cancer were disease-free following vaccine administration [113,114].

#### 3.3.4. HSP70 in OS

HSP70 is significantly overexpressed in OS cell lines compared to human primary cultures of osteoblastic cells [115]. This overexpression is also observed in OS patients’ tissues compared to the adjacent muscle [116]. Beyond its role over heat shock response and protein homeostasis, HSP70 is involved in different mechanisms promoting tumor development in OS, such as cell proliferation, apoptosis, and drug resistance (Figure 3). Triptolide, a bioactive compound that downregulates expression of HSP70 and MKP-1 (Mitogen-activated protein kinase-1, a protein phosphatase that is an endogenous MAPK deactivator), significantly reduces U2OS and MG63 cell proliferation in a dose-dependent manner [117]. Moreover, HSP70 protects OS cell viability thanks to its anti-apoptotic role. Gennaro et al. demonstrate that HSP70 interacting with one isoform of its co-chaperones, BAG1S, mediates survival of OS cells expressing oncogenic MYC. MYC is indeed overexpressed in OS cell lines compared to osteoblasts, as well as in OS patient tissue [118]. The HSP70/BAG1S complex stabilizes anti-apoptotic proteins such as glucocorticoid receptor (GCR), X-linked inhibitor of apoptosis protein (XIAP), and rapidly accelerating fibrosarcoma 1 (RAF1) in OS [119].

HSP70 anti-apoptotic activity promotes drug resistance mechanisms in OS. To illustrate, Baicalein, a new drug that shows promising anti-cancer effects against a wide range of tumors (pancreatic, colorectal and breast cancer), upregulates HSP70 in OS cells. HSP70 decreases the sensitivity of OS cells to Baicalein by preventing apoptosis via the activation of the PI3K/AKT and MAPK/ERK pathways. Thus, the over-expression of HSP70 seems to induce Baicalein resistance in OS cells [120]. Moreover, along with miR-223 and JNK/JUN, HSP70 forms a feedback loop to modulate resistance to CIS in OS cells. While miR-223 expression was usually downregulated in OS cells, miR-223 overexpression enhanced the cellular effects of CIS. HSP70 is a direct target of miR-223 in OS as miR-223 may regulate HSP70 protein levels through binding to HSPA1A 3′UTR. Downstream, HSP70 regulates JNK signaling of which JUN is the main transcription factor. JUN, in turn, is able to bind the promoter region of miR-223, promoting its transcription. HSP70 thus participates in the CIS resistance mechanism in OS cells [121,122].

HSP70 is also an interesting biomarker for OS, as its high expression in biopsies is correlated with poor prognosis for patients [64,74,116]. For this reason, HSP70 may be used as a prognostic marker and a therapeutic target in OS.

#### 3.3.5. Targeting HSP70 in OS

As HSP70 is involved in many mechanisms promoting tumorigenesis in OS, its inhibition could be considered to be a promising therapeutic approach. However, there are not many molecules tested in OS that directly target HSP70.

VER-155008 is an ATP mimetic that binds to the nucleotide binding domain of HSP70, inhibiting its interaction with the substrate binding domain [123]. Treatment with VER-155008 decreases cell proliferation and increases apoptosis in canine OS cell lines [124]. However, treating OS cells with doxorubicin did not enhance the effects of VER-155008, probably due to the rapid compensation mechanisms following HSP70 inhibition, such as HSP70 re-expression and upregulation of the expression of GRP78, a major endoplasmic reticulum chaperone protein [124].

### 3.4. HSP90

#### 3.4.1. Structure

The structure of HSP90 (HSPC) is composed of 3 different domains: an N-domain, a middle domain and a carboxy-terminal domain [11]. The amino terminal region, called the N domain, contains an ATP- and drug-binding site and patterns that interact with co-chaperone proteins. The middle domain provides docking sites for client proteins and co-chaperones, participating in the formation of active ATPase. The carboxy-terminal domain contains a dimerization motif, a second drug binding region and interaction sites for other chaperones [125] (Figure 2). Dimerization is essential for HSP90 chaperone functions [126]. There are different forms of HSP90, encoded by genes located in different chromosomes, depending on its sub-cellular location. HSP90 alpha and beta are located in cytoplasm, GRP95 in the endoplasmic reticulum, and TRAP1 exerts its functions in mitochondria [127].

#### 3.4.2. Regulation

There are different levels of regulation involved in HSP90 functions. HSP90 expression depends on the binding of HSFs to HSE in the HSP promotor region, stimulating action of RNA polymerase II on the coding region of the HSP90 gene [125]. Post-translational modification (PTM) of HSP90 isoforms also modulates their chaperone functions by modifying their binding site activity [21].

PTM depends on its isoforms. Cytoplasmic isoforms undergo phosphorylation, acetylation, SUMOylation, methylation, ubiquitinylation and S-nitrosylation [128]. For the endoplasmic reticulum form, GRP94, glycosylation, phosphorylation and acetylation have been observed [129]. TRAP1, the mitochondrial homologue, can be phosphorylated by PINK1 kinase [130,131]. Co-chaperones represent the most influential layer in HSP90 regulation. HOP and HSP40 are common co-chaperones with HSP70 as they promote HSP70 and HSP90 interaction. Other HSP90 co-chaperones play a part in the pro-tumoral role of HSP90, such as WISp39, which regulates p21 stability, or Aha1, which stimulates its ATPase activity. The co-chaperone CHIP is also involved in unfolded protein degradation and Sgt1 participates in HSP90 client recruitment [127].

#### 3.4.3. The Role of HSP90 in Cancer

The different isoforms of HSP90 are differentially involved in tumorigenesis processes. Cytosolic HSPs are mainly involved in the folding and maturation of several cancer proteins which are essential for cancer development and progression [132]. As examples, some cytosolic HSP90 clients are involved in uncontrolled cell proliferation, such as BCR-ABL, mutant TP53, mutant BRAF, AKT, Cyclin D1, and CDK4 [133]. Others are involved in anti-apoptotic effects, such as NF–kB and Apaf-1. Some client proteins of HSP90 are also involved in invasion and metastasis, such as MMP2 or c-MET, or in immortalization, such as telomerase, or angiogenesis induction, such as HIF-1 alpha, VEGFR or genome instability, and mutations, such as FANCA or MAFG [134].

GRP94 is involved in the quality control of the ER, by chaperoning several proteins involved in carcinogenesis, like toll-like receptors (TLRs), the Wnt co-receptor LRP6, or the insulin-like growth factor (IGF). However, GRP94 can also induce an immune response against tumor cells through cross-presentation of tumoral antigenic peptides and T-cell activation [135]. TRAP1 expression is elevated in many cancers and is described as participating in the carcinogenesis of many tumors, in particular by interfering with aerobic glycolysis through inhibition of succinate dehydrogenase. TRAP1 also supports cell survival by hindering ROS accumulation and maintains protein homeostasis by regulating de novo protein synthesis and protein degradation [136].

#### 3.4.4. HSP90 in OS

HSP90 plays an oncogenic role in OS and is involved in significant cancer hallmarks such as cell proliferation, apoptosis and drug resistance (Figure 3) [137]. HSP90 enhances OS cell proliferation and migration. Liu et al. showed that HSP90 inhibition suppresses AKT1 phosphorylation, decreases ki-67 and Vimentin protein levels, and enhances the protein level of p21 and E-cadherin [138]. Thus, HSP90 and its co-chaperone Aha1 are described as upregulating isocitrate dehydrogenase 1 (IDH1) and enhancing the metabolic activity of OS cells. IDH1 upregulation by HSP90/Aha1, promotes tumor growth and metastasis in vivo in a xenograft model of OS [139]. Moreover, perifosine, an Akt inhibitor known to block Akt/mTOR complex 1 (mTORC1) signaling, leading to apoptosis, was also shown to inhibit survivin expression by disrupting its interaction with HSP90 in OS cells, thus increasing apoptosis, as shown by an increase of cleaved caspase-3 expression and c-Jun activation [140]. Different studies describe HSP90 involvement in OS cell apoptosis. Yang et al. demonstrated that HSP90 regulates OS cell apoptosis by targeting the p53/T cell factor/lymphoid enhancer-binding factor (TCF-1) mediated transcriptional network [141]. HSP90 also indirectly regulates expression of RUNX2, controlling OS cell apoptosis via the AKT/GSK-3β/β-catenin signaling pathway. HSP90 inhibition in OS cells leads to AKT downregulation that decreases the p-GSK-3β/GSK-3β ratio, thus downregulating β-catenin expression. Consequently, downstream effectors of β-catenin, RUNX2 and cyclin D1 are not expressed, thereby leading to caspase-3 mediated apoptosis in OS cells [142]. HSP90 inhibition impoverishes cells from different proteins, playing an anti-apoptotic role such as AKT and IGF1R in OS cells [143]. Blocking HSP90 induces autophagy in OS cell lines through the PI3K/mTOR pathway inhibition. HSP90 inhibition using geldanamycin affects the Akt/mTOR signaling pathway by inhibiting the phosphorylation of the downstream effectors of mTOR in OS cells. Combining an HSP90 inhibitor with 3-methyladenine (3-MA), an inhibitor of autophagy, induced higher apoptosis than geldanamycin alone. [144,145].

HSP90 is also involved in many treatment resistance mechanisms in OS. Conventional chemotherapy drugs such as DOX, CIS and MTX induce HSP90 up-regulation in human OS cells leading to resistance, while HSP90 inhibition restores the sensitivity of OS cells to chemotherapy both in vitro and in vivo [145]. As an example, TRAP1 protects OS cells from stress-induced DNA damage and apoptosis. In fact, an inverse correlation between induction of apoptosis and TRAP-1 levels have been observed in Saos-2 cell lines [146].

Thus, because of its pleiotropic roles in OS development and progression, HSP90 is an interesting molecular target. Its advantage as a biomarker has also been highlighted for its extracellular form. The presence of anti-HSP90 antibodies significantly correlates with a better response to neoadjuvant chemotherapy, whereas their absence is correlated with the development of metastasis in OS patients [147].

#### 3.4.5. Targeting HSP90 in OS

There are many molecules that target HSP90 both in vivo and in vitro in OS, some of which have a therapeutic effect against the biological activity of OS. Tanespimycin (17-AAG) is an analogue of geldanamycin but with lower toxicity. In general terms, 17-AAG inhibits HSP90 by occupying its ATP binding pocket, thus altering the expression level of HSP90 client proteins such as mutant p53, Akt, telomerase, CDK4 or BCR–ABL. 17-AAG inhibits the properties and chemoresistance of OS stem cells by repressing the GSK3β/Hedgehog signaling pathway [148,149]. Moreover, 17-AAG has been tested in phase I pharmacokinetic and pharmacodynamic clinical trial on pediatric patients with recurrent or refractory solid tumors, including OS. As 17-AAG did not show any toxicity for patients, pediatric studies of 17-AAG combined with conventional chemotherapies and other molecular targets are planned [150]. Ory et al. demonstrated that PF4942847, a synthetic HSP90 inhibitor, has superior potency in vitro in a panel of OS cell lines compared to 17-AAG, on the inhibition of cell survival, induction of apoptosis, delayed tumor growth and reduced development of pulmonary metastases in a xenograft model of OS [137]. SNX-2112, a selective synthetic HSP90 inhibitor exerts downstream effects on HSP90 client proteins and demonstrates anti-tumoral activity by reducing AKT1 and C-Raf levels and PARP cleavage induction in OS cell lines [151]. STA-1474 is a prodrug of ganetespib, a small synthetic molecule that inhibits HSP90. STA-1474 induces tumor regression, caspase-3 activation and downregulation of pMet/Met and p-Akt/Akt in an OS xenograft model [152]. Ganetespib combined with the mTOR inhibitor sirolimus have been tested in the phase I/II SARC023 clinical trial on patients with sarcomas (NCT02008877). However, no responses were observed [153].

### 3.5. The Limitations of HSP Targeting in OS

We have observed that HSP27, HSP60, HSP70 and HSP90 play a major role in OS development and can be used as potential clinical biomarkers for diagnosis and prognostic outcomes for patients. Moreover, we have observed that HSPs have pro-proliferative and anti-apoptotic effects, leading to drug resistance in OS (Figure 3). However, only 17-AAG has demonstrated significant relevance for its therapeutic effects in OS. There are several reasons that could explain why HSP inhibitors have controversial effects. First, inhibition of HSP activities is not specific to cancer cells and severely affects normal cells. It has also been observed that inhibition of a single HSP often leads to induction of another single HSP. As an example of this compensation mechanism, HSP90 inhibition with 17-AAG increases HSP70 expression in OS cell lines [149]. Therefore, combining different HSP inhibitors could be interesting in OS to hijack compensation mechanisms. Another approach currently attracting major interest is to target HSF1.

## 4. Involvement of HSF1 in Carcinogenesis and Therapeutic Benefits

### 4.1. Structure

HSF1 belongs to the Heat Shock Factor family with the proteins HSF2, 4, 5, X and Y, all involved in the response to stress. HSF1 is a 57 kDa protein composed of 4 functional domains. At the N-terminal, there is first a DNA binding site that makes it possible to recognize repeated and inverted 5′-nGAAn-3′ consensus sequences, called the Heat Shock Element (HSE). HSF1 also contains two leucine zippers allowing oligomerization between the different monomers. In the absence of stress, these two domains interact to regulate the activation of HSF1 negatively. One regulation domain is also present, the site of the post-transcriptional regulation of HSF1, which allows its activation or repression by phosphorylation, acetylation or SUMOylation. Finally, the C-terminal part contains an activation domain directing HSF1 to its different targets [154] (Figure 2).

### 4.2. Regulation

As previously discussed, HSF1 is mainly regulated by protein–protein interactions and PTMs. HSPs inhibit HSF1 activity. This self-regulation system makes possible a correlation between HSF1 activation and cellular stress level, guaranteeing proteostasis (Figure 1). But HSF1 activation is not only dependent on this feedback mechanism. In fact, hyperphosphorylation of HSF1 is essential for its activation. Phosphorylation on serine 326 and 230 activates HSF1 transcriptional activity [155,156], while other phosphorylations repress its activity. This is the case of serine 303 phosphorylation by GSK3 and serine 307 by ERK1 [157,158]. Ser121 phosphorylation by MAPKAPK2 in the HSF1 DNA binding site diminishes its DNA binding activity and promotes its interaction with HSP90 [159]. HSF1 protein partners are able to modulate its transcriptional activity. This is the case of sirtuin deacetylase, which may deacetylate lysines 80 and 118 of HSF1 to extend its DNA binding [160,161]. Also, the ATF1 protein behaves as a co-repressor of HSF1 by inhibiting its interaction with DNA. This phenomenon has been observed after a heat shock where the interaction between ATF1 and HSF1 allows the recruitment of p300/CBP, leading to the acetylation of HSF1 [162]. Moreover, during cellular stress, HSF1 can recruit SWI/SNF (SWItch/Non-Fermentable Sucrose) which is an ATP-dependent chromatin remodeling complex that facilitates the access of HSF1 to DNA [162].

### 4.3. The Role of HSF1 in Cancer

It has long been accepted that the involvement of HSF1 in cancer is limited to its role in the stress response. However, Dai et al. showed that HSF1 has a more direct involvement in carcinogenesis. They observed in mice mutated on *tp53* (the most frequent mutation in OS), that inhibition of HSF1 significantly decreased tumor incidence. Moreover, mice mutated on *tp53*, expressing homozygous HSF1 (HSF1+/+), developed sarcomas in 70% of cases, compared to *tp53* mutated mice with mono-allelic HSF1 invalidation (+/−) that develop sarcomas in only 30% of cases [163].

Beyond its involvement in carcinogenesis in mice, it has been shown that inhibition of HSF1 significantly reduces tumor proliferation and survival [163]. However, overexpression of HSF1 in healthy cells does not lead to development of a malignant phenotype [164]. These data demonstrated that HSF1 is not an oncogene but a protein that facilitates tumor development in a specific context (e.g., mutation of the *TP53* gene), thus defining the concept of “non-oncogenic addiction”. Thus, HSF1 involvement in carcinogenesis comes from the hijacking of its primary function by tumor cells, notably through its overactivation by the RAS/MAPK pathway or the stress environment induced by the TP53 mutation [8]. It has been shown that HSF1 can play a role in cancer promotion both indirectly via the regulation of HSPs and directly by regulating pro-tumoral gene expression.

Many studies have described HSF1 overexpression in several types of cancer cells (e.g., breast, prostate, colorectal) although its expression does not always correlate with HSP expression [165]. Moreover, inhibition of HSF1 does not lead to a decrease in the expression of HSPs in certain types of cancer, such as prostate cancer [165]. These observations suggest that HSF1 can regulate genes other than those involved in the stress response. Similarly, Mendillo et al. showed through ChIP-Seq and RNA-seq experiments performed on a broad panel of malignant cells (breast, colon, lung) that HSF1 regulates the expression of genes that are not involved in the stress response in healthy cells. These genes involve HSF1 in many biological functions supporting malignancy such as protein translation, cell cycle progression, DNA repair, chromatin remodeling, and metabolic energy [166].

This pro-tumor role of HSF1 has been observed in myeloma and cervical cancer. This is notably the case in myeloma, where HSF1 inhibition using RNA interference induces apoptosis [167]. An aptamer RNA inhibiting HSF1 binding to HSE induces apoptosis in HeLa cell lines [168]. HSF1 is also implicated in resistance mechanisms to chemotherapy by inducing the activity of the ATP binding cassette ABCB1 (MDR1). The MDR1 pump generates an efflux of DOX and paclitaxel, which leads to resistance to chemotherapy [169,170,171].

### 4.4. HSF1 in OS

Limited data are available regarding HSF1 involvement in OS. Zhou et al. observed from 65 OS patients that high expression of HSF1 in tumoral tissue is correlated with poor overall and disease-free survival [172]. Inhibition of HSF1 using RNA interference decreases the proliferation, migration, and invasion of OS cells. HSF1 is also involved in ZOL resistance mechanisms in OS cells through MDR1 regulation. In fact, ZOL induced HSF1 activity, thus increasing MDR1 expression. It has been shown that the MDR1 promoter contains HSE elements [170,173]. Thus, because of its central role in cancer biology, HSF1 is an interesting therapeutic target in the treatment of OS.

### 4.5. The Advantages of Direct Targeting of HSF1 in Oncology

Numerous studies have shown the advantages of HSF1 inhibition in cancer by describing its anti-tumor effects [163]. The increasing number of studies on this subject has been accompanied in recent years by the discovery of several natural or synthetic molecules capable of inhibiting HSF1 activation and its binding to DNA.

The first HSF1 inhibitor molecule discovered is quercetin, a natural compound of the flavone family [154,174]. Quercetin inhibits HSF1 binding to DNA HSE motifs in vitro and in vivo in COS-7 and COLO320DM cells. In addition, quercetin decreases the level of HSF1 phosphorylation in cells undergoing heat shock but not in normal cells. Thus, quercetin decreases HSP expression by inhibiting both the expression and phosphorylation of HSF1 [175]. Quercetin also significantly decreases migration and invasion in OS cell lines, as shown by a reduction in the formation of lung metastases and tumor proliferation in a mouse model of OS [176].

2,4-Bis(4-hydroxybenzyl)Phenol is a natural HSF1 inhibitor isolated from herbs. This compound induces dephosphorylation of HSF1 in serine 326. Through this, 2,4-Bis(4-hydroxybenzyl)Phenol reduces HSF1 stability and consequently its binding to DNA. This molecule has shown synergistic anticancer effects with conventional chemotherapies such as paclitaxel and CIS in lung tumor cells [177].

A synthetic inhibitor, PW3405, inhibits HSF1 phosphorylation, resulting in decreased expression of HSPs. This molecule has shown efficiency at nanomolar concentrations with low toxicity in the prostate, pancreas, lungs, and adenocarcinomas [178].

## 5. The Preclinical and Clinical Advantages of Inhibiting HSPs in OS

HSP27, HSP60, HSP70 and HSP90 chaperone oncogenic driver clients in OS such as BCR/ABL or TP53, and are involved in different pro-tumoral mechanisms such as proliferation and migration enhancement, apoptosis inhibition and conventional chemotherapy resistance mechanisms. As they are overexpressed in many cancers, including OS [14,15], inhibition of HSPs has been studied to decrease tumor progression. All of the molecules tested to inhibit HSPs in OS are summarized in Table 1. To date, HSP90 is the most targeted HSP in OS. All forms of cancer combined, 17 HSP90 inhibitors have been evaluated in clinical trials [179]. However, none has yet been approved by the FDA as a monotherapy for cancer. Geldanamycin is in fact the first molecule used to target chaperones. It failed in its clinical trial and did not progress beyond Phase I because of its cardiac, ocular and hepatic toxicities [179]. Geldanamycin analogues such as 17-AAG demonstrate less toxicity and have successfully passed Phase I pharmacokinetic and pharmacodynamic study in pediatric patients with recurrent or refractory solid tumors such as OS [150]. Tanespimycin (17-AAG) is currently being evaluated in phase I clinical trial with pediatric patients with recurrent or refractory leukemia or solid tumors (NCT00093821).

HSP27 inhibitor (KRIBB-3), HSP70 inhibitor (VER-155008) and HSP90 inhibitor (PF4942847, ganetespib, SNX-2112) have shown promising effects in vitro or in vivo in OS. However, as HSPs are highly regulated molecules, compensatory mechanisms are both inevitable and an obstacle to the development of therapies based on their simple inhibition. HSF1 can compensate for the inhibition of HSPs by strongly re-expressing the latter. This is why the HSF1 inhibitor quercetin could be interesting in combination with HSP inhibitors in OS.

## 6. Conclusions

This review manuscript highlighted the leading role played by HSPs in OS cell survival. Because of their pro-proliferative, anti-apoptosis and pro-resistance role, HSPs are interesting prognosis biomarkers and therapeutic targets for OS cell lines. Their main role in proteostasis in healthy cells and their highly compensatory effect prevent them from being considered as a therapy based on their own inhibition. Targeting their main regulator, HSF1, may be a therapeutic solution. Although direct targeting of HSF1 shows promising anti-tumor effects, its central role in several biological processes is a barrier to the development of therapies based on its simple inhibition. While HSR promotes carcinogenesis, it remains an essential mechanism for maintaining cellular proteostasis in the face of various environmental aggressions under physiological conditions. Moreover, it has been shown that HSF1 is involved in functions that go far beyond the response to stress. In particular, HSF1 is involved in the development, reproduction and regulation of the energy metabolism [25]. Better understanding of the molecular mechanisms implicating HSF1 and HSPs in the various pro-oncogenic functions related to them would make it possible to develop therapies aimed at inhibiting only these functions without affecting their physiological role.

## Figures and Tables

**Table 1:** Overview of HSPs and HSF1 involvement in tumorigenesis and of their inhibitors tested in OS (nd: no data).

HSP Targeted	Effects on Tumorigenesis in OS	Potential as Biomarker in OS	Molecule Targeting and Clinical Relevance in OS
**HSP27**	Tumor growth: Promote OS cell proliferation [66] Metastasis: Decrease cell migration under hyperthermia [67] Relapse: Correlated with expression level in tumor [73]	Overexpression: associated with poor prognosis for patients and metastasis [64,73,74,75] p-HSP27 expression: Biomarker to define autophagy response to chemotherapy [77]	KRIBB-3 [78,79] Inhibition: Direct Effects: (in vitro/in vivo) Diminish resistance mechanisms/ Pro-apoptosis [77,78] Bufalin [69] Inhibition: Indirect, mediated by proteasome Effects: (in vitro) Pro-apoptosis
**HSP60**	Tumor growth: Promote OS cell proliferation [92] Metastasis: nd Relapse: nd	Overexpression: Not correlated with clinical outcomes [64,74] Anti-HSP60 antibodies: Presence could be used as diagnosis [91]	Geldanamycin [94] Inhibition: Indirect, Loss of HSP60 hyperacetylation Effects: (in vitro) Anti-proliferation/Pro-apoptosis
**HSP70**	Tumor growth: Promote OS cell proliferation [117] Metastasis: nd Relapse: nd	Overexpression: associated with poor prognosis for patients [64,74,116]	VER-155008 [124] Inhibition: Direct Effects: (in vitro in canine cell lines) Pro-proliferation/Pro-apoptosis
**HSP90**	Tumor growth: Promote tumor growth in vivo [137] Metastasis: Promote metastasis formation in vivo [137,139] Relapse: nd	Overexpression: Not correlated with clinical outcomes [64] Anti-HSP90 antibodies: Presence is correlated with a better response to neo-adjuvant chemotherapy and absence is correlated with metastasis development [147]	17-AAG [148,149] Inhibition: Direct Effects: (in vitro/Phase I Clinical Trial) Pro-differentiation/Anti-chemoresistance PF4942847 [137] Inhibition: Direct Effects: (in vitro/in vivo) Anti-proliferation/Pro-apoptosis Ganetespib [153] Inhibition: Direct Effects: (in vivo) Tumor regression SNX-2112 [151] Inhibition: Direct Effects: (in vitro) Anti-proliferation/Pro-apoptosis
**HSF1**	Tumor growth: Promote OS cell proliferation [172] Metastasis: Promote OS cell migration and invasion [172] Relapse: nd	Overexpression: associated with poor overall and disease-free survival [172]	Quercetin [176] Inhibition: Direct Effects: (in vitro/in vivo) Anti-migration, anti-invasion, diminish tumor proliferation
